# Contributions of representing the elements of nursing practice in the ISO 18.104:2023 standard: a theoretical study

**DOI:** 10.1590/1980-220X-REEUSP-2023-0358en

**Published:** 2024-04-05

**Authors:** Marcia Regina Cubas, Rudval Souza da Silva, Cândida Caniçali Primo, Marcos Antônio Gomes Brandão, Nuno Damácio de Carvalho Félix, Rodrigo Jensen

**Affiliations:** 1 Pontifícia Universidade Católica do Paraná, Escola Politécnica, Programa de Pós-Graduação em Tecnologia em Saúde, Curitiba, PR , Brazil.; 2 Universidade do Estado da Bahia, Senhor do Bonfim, BA, Brazil.; 3 Universidade Federal do Espírito Santo, Programa de Pós-Graduação em Enfermagem, Vitória, ES, Brazil.; 4 Universidade Federal do Rio de Janeiro, Escola de Enfermagem Anna Nery, Rio de Janeiro, RJ, Brazil.; 5 Universidade Federal do Recôncavo da Bahia, Santo Antônio de Jesus, BA, Brazil.; 6 Universidade de São Paulo, Escola de Enfermagem, São Paulo, SP, Brazil.

**Keywords:** Standardized Nursing Terminology, Nursing Process, Nursing Records, Electronic Health Records, Terminologia Padronizada em Enfermagem, Processo de Enfermagem, Registros de Enfermagem, Registros Eletrônicos de Saúde, Terminología Normalizada de Enfermería, Proceso de Enfermería, Registros de Enfermería, Registros Electrónicos de Salud

## Abstract

**Objective:**

To reflect on the contributions of representing nursing practice elements in the ISO 18.104:2023 standard.

**Method:**

This is a theoretical study with standard analysis. Categorical structures were described to represent nursing practice in terminological systems and contributions identified in the parts of the version were analyzed.

**Results:**

There is innovation in the inclusion of nurse sensitive outcomes, nursing action, nursing diagnosis explanation as an indicator of nursing service demand and complexity of care, representation of concepts through mental maps and suggestion of use of restriction models for nursing actions. It describes that the Nursing Process is constituted by nursing diagnosis, nursing action and nurse sensitive outcomes.

**Final considerations:**

Indicating a nursing diagnosis as an indicator will bring benefits for knowledge production and decision-making. Although care outcomes are not exclusive responses to nursing action, the modifiable attributes of a nursing diagnosis generate knowledge about clinical practice, nursing action effectiveness and subjects of care’ health state. There is coherence in understanding the Nursing Process concept evolution.

## RESUMO

**Objetivo:**

Refletir sobre as contribuições da representação dos elementos da prática de enfermagem na norma ISO 18.104:2023.

**Método:**

Estudo teórico com análise da norma. Foram descritas estruturas categoriais para representação da prática da enfermagem nos sistemas terminológicos e analisadas as contribuições identificadas nas partes da versão.

**Resultados:**

Há inovação na inclusão do resultado sensível à ação da enfermeira, explicitação do diagnóstico de enfermagem como indicador da demanda de serviços e complexidade de assistência, representação dos conceitos por mapas mentais e sugestão do uso de modelos de restrição para ações de enfermagem. Descreve que o Processo de Enfermagem é constituído pelo diagnóstico, ação e resultado sensível à ação da enfermeira.

**Considerações finais:**

A indicação do diagnóstico de enfermagem como um indicador trará benefícios para produção de conhecimento e tomada de decisão. Embora os resultados do cuidado não sejam respostas exclusivas a uma ação da enfermeira, os atributos modificáveis de um diagnóstico de enfermagem geram conhecimentos sobre a prática clínica, a eficácia das ações de enfermagem e o estado de saúde dos sujeitos de cuidado. Há coerência na compreensão da evolução do conceito de Processo de Enfermagem.

## RESUMEN

**Objetivo:**

Reflexionar sobre los aportes de representar los elementos de la práctica de enfermería en la norma ISO 18.104:2023.

**Método:**

Estudio teórico con análisis de la norma. Se describieron estructuras categóricas para representar la práctica de enfermería en sistemas terminológicos y se analizaron las contribuciones identificadas en las partes de la versión.

**Resultados:**

Hay innovación en la inclusión del resultado sensible a la acción del enfermero, explicación del diagnóstico de enfermería como indicador de demanda de servicios y complejidad del cuidado, representación de conceptos a través de mapas mentales y sugerencia del uso de modelos de restricción para las acciones de enfermería. Describe que el Proceso de Enfermería está constituido por el diagnóstico, la acción y el resultado sensible a la acción del enfermero.

**Consideraciones finales:**

La indicación del diagnóstico de enfermería como indicador traerá beneficios para la producción de conocimiento y la toma de decisiones. Aunque los resultados del cuidado no son respuestas exclusivas a la acción del enfermero, los atributos modificables de un diagnóstico de enfermería generan conocimiento sobre la práctica clínica, la efectividad de las acciones de enfermería y el estado de salud de los sujetos del cuidado. Hay coherencia en la comprensión de la evolución del concepto de Proceso de Enfermería.

## INTRODUCTION

Since 1947, the International Organization for Standard (ISO) has acted as a non-governmental organization that encourages discussions and publications of standards that promote quality and safety for the development and manufacture of goods, products and services. The organization counts on the participation of researchers and experts from different countries in favor of a consensus on standardization.

ISO 18.104, a reference for representing nursing practice in computer terminological systems, is in its third version. The first version was published in 2003^(1)^, the second in 2013 and in Brazilian Portuguese in 2014^(2)^. The draft version of the third was made available at the end of 2022 and the official version at the end of 2023^(3)^. ISO 18.104:2023 presents the categorical structure for nursing practice computational representation and describes aspects related to specialized language systems or terminological systems, which include in a special way the three elements of nursing practice: nursing diagnosis; nursing action; and nurse sensitive outcomes.

ISO 18.104:2023 recognizes nurses’ work as a member of the interdisciplinary team so that the categories and subcategories described in the standard are applicable to other clinical disciplines, supporting a better representation of knowledge base of each discipline and the construction of management protocols service using standardized terminology^(3)^.

Using regulations that guide standardization in the construction of nursing diagnosis, outcome and action statements contributes to genuine and unambiguous language applied in different care scenarios, playing an essential role for retrievable and interoperable electronic documentation. The fact that there is standardization in the record does not hamper nurses’ practice, but allows the documentation of relevant and mandatory information related to the elements of the Nursing Process that represent the health situation of persons, families or communities assisted. Therefore, nurses must be given the possibility of recording narrative texts, with details that address the uniqueness of people, families or communities under their care^(4)^.

To allow interoperability and retrieval of information from electronic records, using the Systematized Nomenclature of Medicine - Clinical Terms (SNOMED CT)^(5)^ has been recommended in many countries, which is a reference terminology. Using reference terminology does not imply the disappearance of interface terminologies used by specific professional domain. Interface terminologies such as the International Classification for Nursing Practice (ICNP^®^), NANDA International Inc. (NANDA-I), the Nursing Outcomes Classification (NOC) and the Nursing Interventions Classification (NIC) must be developed and used in practice^(4)^. Ideally, reference and interface terminologies work together so that nursing practice data is represented in health information systems^(6)^.

Given the relevance of ISO 18.104:2023 for elaborating statements that represent nursing practice and for organizing concepts capable of explaining the nursing knowledge domain’s contribution in retrievable databases and, consequently, for the production of science, this article presented a theoretical study that discusses the categorical structure of the elements of nursing practice, with a view to the innovative aspects of the standard based on the description of the parts that make up its text.

This article aimed to reflect on the contribution of representing the elements of nursing practice in ISO 18.104:2023.

## METHOD

This is a theoretical study based on ISO 18.104: 2023 analysis in health informatics, which deals with the representation of categorical structures of nursing practice in terminological systems. Researchers’ and authors’ experiences on the subject, supported by theoretical and normative references that support the development and evolution of terminological systems for nursing practice, allowed to prepare the study.

ISO 18.104, draft version, acquired in 2022, was read by all authors in isolation, and the main points were highlighted for collective discussion in order to understand the version’s innovations and, based on them, analyze the contributions identified in each part of the text, which is organized into a foreword, introduction, eight chapters and two annexes. The official 2023 version, acquired after its release, was analyzed and identified additions were incorporated into the text.

Due to the type of article, opinion from a Research Ethics Committee was not required. The authors of the article are responsible for the integrity of the content and are committed to good practices for publication.

### Innovations and Contributions of the Standard Introductory Content

Foreword generically describes how committees for developing ISO standards are established and informs that the Technical Committee on health informatics responsible for preparing ISO 18.104 is ISO/TC 215. This committee was created in 1998 and has 33 member associations, and until May 2023, was responsible for drafting 228 standards, with 64 in progress. Therefore, it is a consolidated committee.

Introduction presents the justification for developing and updating the standard and describes nursing diagnosis, nursing action and nurse sensitive outcome concepts. It is worth highlighting that the literal translation limits the understanding of this last concept, as it would be “*resultado sensível à enfermeira*”. As the Brazilian Association of Technical Standards (ABNT - *Associação Brasileira de Normas Técnicas*) has not yet made a version available in Brazilian Portuguese, it is understood that nurse sensitive outcome is an outcome resulting from an action carried out by a nurse or, in the case of countries where nursing is carried out by different categories, the most assertive translation would be “*resultado sensível à ação da enfermagem*”. For this study, we chose to use the term “*resultado sensível à ação da enfermeira*” (nurse sensitive outcome), understanding that this professional category, in Brazil, is responsible for determining a nursing diagnosis, prescribing care and nursing assessment.

Introduction highlights that, although the standard is aimed at the nursing domain, the clinical categories and subcategories used are applicable to other disciplines, having characteristics in common with ISO/TS 22.789, with regard to clinical findings, and ISO 1.828, ISO EN 13.940:2015 and the International Classification of Health Interventions, with regard to actions.

It should be noted that the standard states that nursing diagnosis, nursing action and nurse sensitive outcomes concepts constitute the Nursing Process. The Nursing Process is a standard for nursing practice and allows nursing to be distinguished from other professional domains. It is therefore reaffirmed that nursing’s primary identity is care implemented through the Nursing Process, which guides doing and thinking, enabling professional practice documentation^(7)^. There is coherence between the Nursing Process concept evolution, analyzed by Brazilian researchers^(7)^, and the description offered by ISO 18.104:2023, strengthening the use of the standard in teaching, research and extension in nursing.

Nursing diagnosis and action definitions were not modified in the new version, but an important statement was added: a nursing diagnosis is used as an indicator of nursing service demand. This statement has been the subject of recent research, as in a study^(8)^ that sought to analyze the correspondence between nursing diagnosis and care demands, with results indicating that nursing diagnoses have the potential to indicate complexity of care and are convenient for assessing nursing team workload and in a study^(9)^ in which the authors describe that the number of nursing diagnoses demonstrated to be an independent predictor of the effective length of hospital stay and length of stay longer than expected.

Thus, it is important to include the potential use of terminologies in the new version of ISO, especially nursing diagnosis registration, as indicators of nursing service demand and complexity of care. This is a relevant contribution to be considered by researchers when making decisions about the object or establishing a research problem and by managers when analyzing care and management indicators.

Returning to the term “nurse sensitive outcomes”, the standard reinforces the need to differentiate the concept, structure or context between two elements: diagnosis and outcome. In previous versions, nursing outcome was a secondary concept. It could be a goal (expected result) or a new nursing diagnosis identified after carrying out prescribed actions, assessed by the extent of changes^(2)^. Its categorical structure was identical to that of the nursing diagnosis; therefore, no emphasis was placed on representing the concept structure. The class diagram that represented a nursing diagnosis was indicated to represent a nursing outcome.

At this point, it is worth reflecting on the two concepts. ISO 18.104:2023 describes that data collection supports the assessment and that the data must be interpreted by nurses, contemplating a dialogue with the subject of care, enabling a conclusion, i.e., a clinical judgment^(3)^. As the standard is (or will be) used in several countries, the term “clinical judgment” is understood as a nursing diagnosis, nursing problem or nursing need. Given this, ISO 18.104 uses “nursing problem” or “nursing need” as synonyms for the preferred term “nursing diagnosis”, which is defined as a designation given to an assessment finding, event, situation or health problem to indicate that it is considered by nurses and subjects of care as worthy of attention^(3)^.

In turn, nurse sensitive outcome is defined as an observable and/or measurable state, directly or indirectly, in relation to a specificity of care and its relationship with the environment at a given point in time and documented to interconnect the objective of a nursing action relevant to a nursing diagnosis^(3)^. The standard highlights that such outcomes are not exclusive to nursing actions related to a nursing diagnosis, but are also expressed based on nursing diagnosis attributes in order to generate new knowledge about clinical practice, nursing action effectiveness and patients’ health state.

It should be noted that the standard presents the preferred term - nursing diagnosis -, but states that substitute terms can be used - nursing problem or nursing need. If the concept’s maturity is analyzed, countries that already have nursing diagnosis included in legislation and applied in the context of training, continuing education and services should use the preferential term. In turn, it is expected that computational representation can identify expressively the relationship between diagnosis, outcome and action, determining which outcome was more sensitive to nurses.

In version 2023, a paragraph was added about what is not part of the standard’s scope, namely: detailing of categories (or attributes) that make up a nursing diagnosis, nursing outcome and nursing action, nor the detailing of specific terminology; the model for missing, unplanned or unrealized components; diagnoses and actions carried out by nurses who carry out other professional practices; and possible knowledge relationships, such as the causal relationships between concepts. This inclusion is important to establish the standard’s scope and limit.

### Innovations and Contributions of the Standard Chapter Content

The first chapter, called scope, comprises the categorical structure of nurse sensitive outcome. Its content reinforces the dual function of a categorical structure, such as nursing practice analysis and nursing content development in electronic records, clarifying that the standard does not refer to professional activity itself, but to representation of nursing diagnosis, nursing action and nurse sensitive outcome concepts in information systems.

The representation of the set of standard categories is presented by mind maps. This differs from previous versions, in which representation was given by a Unified Modeled Language (UML) class diagram - a form of visual description of the computational data model that presents classes, attributes, operations that the class can use and the relationships between objects. In relation to the draft version, the 2023 version separates mind maps in a chapter called “semantic links” (chapter four). The inclusion of this chapter provided a more didactic structure for the standard. However, in this article, the chapters referring to scope and semantic links are analyzed together.

It should be noted that ISO does not authorize using representative figures in external publications, unless explicit justification and official authorization from the association. Access to the standard occurs by purchasing a copy on the entity’s website, and ISO offers free access to part of the standard’s content, where one can analyze the old and current representation.

In the first mind map, nursing practice is a central element and is directly linked to nine general terms, their categories and subcategories, all of them defined in the chapter called “Terms and definitions”. The general terms with the least relational complexity are: nurse sensitive outcome, which does not have a subcategory; diagnosis, which is related to a clinical finding; goal, that is related to time frame and focus; target, which is related to location and clinical record; event objective (goal), which is related to usage scenario; and preferences, which are related to allergies, diet intolerance and choice of care or service option. The term with medium relational complexity is “subject of care”, which has a relationship with an identifier, the type of subject (individual, group/family or community) and with associated individuals, which in turn has the subject’s relationship as a subcategory care for other subjects.

Two terms whose description in the mind map is of greater relational complexity stand out. The first is “nursing action”, which is related to method, action type, action frequency, technique, tool used, timing and observation (evaluation). This last subcategory is also complex and is related to measure, status, clinical course, severity, site, related event and information from subject of care/carer. The second is outcome causation, which is related to twelve categories: clinical finding; medical diagnosis; treatment impact; confounding factor; supply availability; local environment (including the social determinants subcategory); scheduling issues; tool availability; risk minimization; healthcare provider; human resource management; and self-care behaviour.

A mind map, again, emphasizes nurse sensitive outcome concept as the complexity of the relationships between action and outcome causation will reflect in a greater description of what was modified from a nursing action. When analyzing large databases, these relationships can generate evidence of care. This structure brings an innovative aspect to the standard and allows generating a hypothesis that the assessment of nursing actions that produce sensitive outcomes will be a central and priority theme in research demands in search of evidence.

Such a demand can be identified in recent literature. A Dutch study that explored sensitive outcome use by nurses concluded that there is a lack of information on how nurses can and should be supported by outcome measurement tools^(10)^. In pediatric nursing, a literature review presented a total of 57 sensitive outcomes for use in the specialty^(11)^. The conclusion of both studies highlights the need to reach international consensus to guarantee minimum standards for actions or at least national guidelines. In mental health nursing, a systematic literature review assessed outcomes that could generate quality indicators, concluding that the studies were of variable quality and that the seven indicators proposed at the conclusion of the review should be integrated into administrative data^(12)^.

From these three examples, it is possible to reflect that knowledge gap is due to its complexity, i.e., it is necessary to research more about which indicators will be used to analyze sensitive outcomes, and that the indicators cannot be disconnected from administrative-managerial aspects. In this regard, the causal relationships of outcomes proposed by ISO 18.104: 2023 could be an important data recovery instrument, if they are used in their entirety in information and recording systems.

The second chapter lists the standards that are references for the terms and definitions presented in the other chapters. These standards are: ISO 17115:2020 – Representation of categorical structures of terminology; ISO/TS 22789:2010 – Conceptual framework for patient findings and problems in terminologies; and CSN EN 12264:2005 - Categorical structures for systems of concepts, all linked to health informatics. Knowledge, or at least reading, of the listed standards provides greater understanding of the terms defined in ISO 18.104: 2023. Therefore, it is suggested that those responsible for constructing standardized terminologies and incorporating them into electronic records take their content into account.

The third chapter presents terms and definitions. They are divided into subchapters with: 14 general terms; seven categories of health entities for nursing diagnoses; 14 subcategories of healthcare entities applicable to a clinical assessment including nursing diagnoses; two categories and eight subcategories of nursing actions not previously specified; two categories and 13 subcategories of nurse sensitive outcomes.

In version 2023, the term “nurse” was added, which was not defined in previous versions. Standardly, a nurse is a “specially trained individual who provides autonomous, collaborative and holistic healthcare for the subject of care, carers and significant others in response to their health, behavioural, social and physical situation at a point in time.”^(3)^. The three notes of the definition explain that care covers all ages as well as family, groups and communities, sick or well, including midwives and highlighting the provision of support and comfort as a practice of the profession. The definition is directly related to the standard’s understanding of nursing scope, not limiting practice to direct beneficiary of care, but also to their carers and their social environment.

In the representations of nursing diagnosis and nurse sensitive outcomes, major differences from the 2023 version can be identified. Standard developers include direct and indirect relationships to represent the composition of a diagnosis and an outcome in mental maps. The same one presented for nursing practice is used, but with diagnosis or outcome in the center of the map.

In general, a mental map represents the relationship of a nursing diagnosis with 11 terms. A nursing diagnosis is directly linked to observation (evaluation), nursing action and nurse sensitive outcomes. In the new version, it is understood that a nursing diagnosis is related to a clinical assessment, therefore it offers specific subcategories for this relationship. This relationship can be used to teach clinical reasoning and to choose the minimum data to be collected to relate to a more accurate diagnosis.

In turn, nursing action is an activity aimed directly or indirectly at improving or maintaining health state and necessarily depends on a target and is directly related to nurse sensitive outcomes^(3)^. It has eight additional terms, such as service delivery method, action type, action frequency, technique, tool used, timing, location and record.

Nurse sensitive outcome is described as a “state observed and/or measured directly or indirectly concerning a subject of care and their relationship with the environment at a point in time and documented to suit a use case”^(3)^. Representation in a mind map indicates a direct relationship with “outcome causation”, which is described as the way in which one of the subcategories demonstrated to have contributed to modifications of one of the attributes of the action category, resulting in nurse sensitive outcomes. In other words, it reinforces the idea that it is possible to identify which action promoted the sensitive result or which subcategories were most relevant for modifying the initial diagnosis.

In chapter five, aimed at software developers, the categorical structures that support the principles of conformity are exposed based on requirements specified in EN 12.264 and ISO 17.115 as well as the mandatory information to be included. For the nursing domain, especially for nurses who work with information management, the chapter is extremely important as it presents the requirements for including terminologies in computer systems.

Chapters six, seven and eight present the categorical structures to represent nursing diagnoses, nursing actions and nurse sensitive outcomes. For the nursing diagnoses category, the standard reinforces that observation is an action that assesses clinical course, using reflective thinking to identify problems, risks and potentialities, in order to synthesize information from subject of care through a nursing diagnosis^(3)^. It must be expressed by a focus arising from or qualified by observation attributes that originate from information received from a subject of care, their relationship, preferences and mandatory requirements determined by allergies or diet intolerances. Thus, a nursing diagnosis can be represented by any of its categories or attributes (positive or negative), in addition to being possible to include the term “Risk of” or “Potential/Opportunity/Chance of”, when dealing with phenomena/negative or positive judgments, respectively.

As a nominal category, nurse sensitive outcome can be described in the same way as the diagnosis, presented at a subsequent point in time. The 2023 version reinforces that the outcome is not exclusive to nursing actions, however it generates new knowledge about clinical practice, operational effectiveness and health state.

Nursing action is an intentional act applied to one or more targets, influenced by the type of subject and environment. Therefore, an action, represented by verbs or verbal phrases, must be related to a subject and have at least one target, except when it is explicit in the action^(3)^. It is important to highlight that, to record actions, the standard indicates that the verb should not be used in the infinitive, i.e., if action is “to observe” the record must be included as “observation” or, if carried out, “observed”. At this point, the standard does not make it clear how care prescription should be recorded, as the verb is routinely used in the infinitive.

### Innovations and Contributions of the Standard Annex Content

Two extremely important points are presented in the annexes to exemplify its use. Annex A presents the breadth of nursing practice scope, in line with the International Council of Nurses description adopted by the World Health Organization. It confirms that the main focus of nursing is people’s responses to health problems and real or potential life events. It presents the profession as a practice that includes health promotion and disease prevention, which encompasses autonomous and collaborative care aimed at individuals of all ages, families, groups and communities, sick or well, and in all environments, in addition to indicating functions such as promoting a safe environment, research, participation in health policy formulation, care and health system management, and education.

The annex content clarifies and reinforces that practice is governed by specific legislation in each country in which it is carried out, by educational policy, resources and nurse competency. It highlights the statement that, regardless of the context, nurses are responsible to their subjects of care and to regulatory bodies for the judgments and decisions they make, and the actions they take or delegate to others. Thus, it is understood that ISO 18.104:2023 indicates the need for attention to the deontological aspects of the profession and not just the terminological issue.

In the item dedicated to the Nursing Process, the standard highlights nurses’ multidisciplinary action, stating that all health professionals follow a similar reasoning process. For the standard, the Nursing Process consists of nursing assessment, which provides the necessary information to establish agreement with subjects of care and carers about what needs to be done, when and by whom. To interpret the data and identify actions, clinical reasoning and evidence-based guidelines are used, when available, ending with the overall outcome assessment. Nurse sensitive outcomes inform the development of evidence-based guidelines and should allow identification of possible causes of internal and external outcomes. In general, the standard presents the Nursing Process didactically and relatively, which can enhance its use in training and continuing education spaces.

When presenting information models and terminologies, the standard clarifies that the interface between the information model and the terminology incorporated in the system needs to be carefully assessed to minimize the risk of inaccurate communication. Thus, it indicates that using interface terminologies with interoperability with reference terminologies is essential, both due to the use of terminologies with pre-coordinated statements and the use of those that allow post-coordination. It should be remembered that several nursing terminologies are already represented in SNOMED CT, such as ICNP® and the Clinical Care Classification (CCC).

Still in Annex A, the standard offers support for understanding what is involved in nursing assessment, pointing to collaborative practices and advanced practices in some countries. The standard highlights that nursing assessment recording must be done based on what is “worthy of highlighting” by both parties involved – nurse and subject of care. In turn, it reinforces, once again, the idea that a nursing diagnosis should be used to establish goals.

Regarding care plan, Annex A represents the need to use evidence, presenting situations in which action planning is not so explicit (pre-hospital care and emergencies) and possible situations that interfere with operationalization. It highlights that the complexity of a care plan depends on communication between the professional and the person and that the plan format is directly linked to professional regulations of the countries in which practice takes place.

Thus, we verified a specific contribution of using evidence to compose the plan and the analysis of a plan’s adequacy to the needs and context of people, families and communities for whom care will be provided. It is a paradigm shift that places nursing in a decision-making position and, at the same time, a co-participant in the decision of the plan to be executed by a subject of care.

When describing the nursing action in the annex, the emphasis given to models that can identify and suggest actions that should not be performed in a restriction model aimed at the safety of care is considered a contribution. Another relevant point is the discussion about the difference between nursing action and nursing intervention, widely discussed in interface terminologies. It is said that a nursing action encompasses assessment, planning and more direct interventions, therefore the term “nursing action” is more comprehensive than “nursing intervention”.

When subsequent assessments (called progress assessments) are presented, the annex highlights the importance of considering subjects of care’s own assessment, but states that it is necessary to differentiate the nursing actions outcomes from those of other professionals or subjects of care; therefore, outcome indicators and scales are relevant instruments, offering examples from the NOC, the OMAHA system and the International Classification of Functioning (ICF®). Regarding nurse sensitive outcomes, the annex provides practical examples of how to identify them.

Annex B describes a set of explanatory notes aimed at developers of information systems that must be adopted by nurses who participate in electronic record system implementation.

## FINAL CONSIDERATIONS

ISO 18.104:2023 does not refer to professional activity itself, but to the representation of nursing diagnosis, nursing action and nurse sensitive outcome concepts in information systems. Their analysis brought important points for reflection, such as coherence with the understanding of the Nursing Process concept evolution, which in Brazil must be enhanced by reviewing regulations relating to the subject, recently publish by the authority that regulates the exercise of the profession. ISO 18.104:2023 describes that the Nursing Process is constituted by nursing diagnosis, nursing action, nurse sensitive outcomes and nursing practice scope, and this description is coherent with the Nursing Process concept evolution internationally.

Although nursing diagnosis and nursing action definitions have not been modified, the addition of the indication that a nursing diagnosis can be used as an indicator of nursing service demand is an important statement and should bring benefits to knowledge production when data is retrieved from information systems.

Significantly, nurse sensitive outcome concept inclusion stands out, understanding that, although the outcomes may not be exclusive responses to a nurse’s action, the modifiable attributes of a nursing diagnosis can generate knowledge about clinical practice, nursing action effectiveness subjects of care’ health state. The latter, according to the standard, are considered participants, as they help to define diagnosis and care plan. However, it appears that, in Brazil, the role of subjects of care is a distant reality, although it is established in the organic law of the Brazilian Health System.
